# Newborn screening for medium chain acyl-CoA dehydrogenase deficiency in England: prevalence, predictive value and test validity based on 1.5 million screened babies

**DOI:** 10.1258/jms.2011.011086

**Published:** 2011-12

**Authors:** Juliet Oerton, Javaria M Khalid, Guy Besley, R Neil Dalton, Melanie Downing, Anne Green, Mick Henderson, Steve Krywawych, James Leonard, Brage S Andresen, Carol Dezateux

**Affiliations:** MRC Centre of Epidemiology for Child Health, UCL Institute of Child Health, London, United Kingdom; MRC Centre of Epidemiology for Child Health, UCL Institute of Child Health, London, United Kingdom; Royal Manchester Children's Hospital, Manchester, United Kingdom; Evelina Children's Hospital, London, United Kingdom; Sheffield Children's Hospital, Sheffield, United Kingdom; Birmingham Children's Hospital, Birmingham, United Kingdom; St James' University Hospital, Leeds, United Kingdom; Great Ormond Street Hospital for Children, London, United Kingdom; MRC Centre of Epidemiology for Child Health, UCL Institute of Child Health, London, United Kingdom; Research Unit for Molecular Medicine, Aarhus University Hospital, Denmark and Department of Biochemistry and Molecular Biology, University of Southern Denmark, Denmark; MRC Centre of Epidemiology for Child Health, UCL Institute of Child Health, London, United Kingdom

## Abstract

**Background:**

Medium chain acyl-CoA dehydrogenase deficiency (MCADD) is a rare, life-threatening condition. Early diagnosis by screening asymptomatic newborns may improve outcome, but the benefit to newborns identified with variants not encountered clinically is uncertain.

**Objective:**

To estimate, overall and by ethnic group: screen-positive prevalence and predictive value (PPV); MCADD prevalence; proportion MCADD variants detected of predicted definite or uncertain clinical importance.

**Setting:**

All births in areas of high ethnic minority prevalence in England.

**Methods:**

Prospective multicentre pilot screening service; testing at age five to eight days; standardized screening, diagnostic and management protocols; independent expert review of screen-positive cases to assign MCADD diagnosis and predicted clinical importance (definite or uncertain).

**Results:**

Approximately 1.5 million babies (79% white; 10% Asian) were screened. MCADD was confirmed in 147 of 190 babies with a positive screening result (screen-positive prevalence: 1.20 per 10,000; MCADD prevalence: 0.94 per 10,000; PPV 77% [95% CI 71–83]), comprising 103 (70%) with MCADD variants of definite clinical importance (95 white [95%]; 2 Asian [2%]) and 44 (30%) with variants of uncertain clinical importance (29 white [67%]; 12 Asian [28%]).

**Conclusion:**

One baby in every 10,000 born in England is diagnosed with MCADD by newborn screening; around 60 babies each year. While the majority of MCADD variants detected are predicted to be of definite clinical importance, this varies according to ethnic group, with variants of uncertain importance most commonly found in Asian babies. These findings provide support for MCADD screening but highlight the need to take account of the ethnic diversity of the population tested at implementation.

## INTRODUCTION

Medium chain acyl-CoA dehydrogenase deficiency (MCADD) is an autosomal recessive disorder of fatty acid oxidation, affecting between 1 in 10,000 to 27,000 babies of northern European descent.^1–5^ MCADD results in reduced ability to break down fatty acids to meet demands during periods of metabolic stress. Typically, it presents during an intercurrent illness or a period of fasting, with symptoms which can include hypoglycaemia, vomiting and encephalopathy. These may lead to coma and sudden death which can be the presenting feature in a child hitherto thought to be healthy. It has been estimated that up to a quarter of previously undiagnosed children die during their initial acute episode, with a further 16% surviving with a severe neurological disability.^6–10^ Once a diagnosis is made, a high carbohydrate intake is given when at risk, together with avoidance of fasting.^11^

Newborn screening for MCADD measured by tandem mass spectrometry (MS/MS) based on the primary biomarker of octanoylcarnitine (C8)^1^ has been introduced in many countries.^12–14^ The short-term clinical outcome following presymptomatic diagnosis through screening is good,^15,16^ although death and serious decompensation may still occur.^17,18^ However, there is uncertainty as to whether all those detected benefit from screening since homozygosity for c.985A>G – the MCADD genotype most associated with clinically severe disease – is found less often in those detected through newborn screening compared with those presenting clinically (55% and 80% of cases respectively)^2,5,19–27^ and a two- to threefold increase in MCADD diagnoses is reported.^14,28,29^ It is unclear to what extent this difference reflects under-diagnosis of clinically presenting children, reduced penetrance and the detection by newborn screening of a less severe disease that may never present clinically.^3,21,28,30^ Many screen-detected MCADD-associated genotypes have never been found amongst reported symptomatic children.^2^ Furthermore, England and Wales has a diverse ethnic population: over 20% of babies born are from a non-white ethnic origin, with about half being of Asian (Indian subcontinent) ethnicity,^31,32^ where little is known about the spectrum of genotype and any associated disease risk.

It was these considerations, coupled with a lack of high-quality evidence regarding test performance, sample timing, screening cut-off for C8, and the spectrum of mutations in a UK context^33,34^ that led the UK Department of Health and National Screening Committee to commission a pilot screening service and a concurrent research evaluation. We established a collaborative group which designed a large-scale pilot MCADD screening service, and undertook a prospective evaluation of screening test performance and outcome using standardized, quality assured screening and diagnostic protocols and with systematic ascertainment of all new diagnoses.

This pilot screening service was introduced in England in March 2004 and ran for four years. We report here the performance of this service, notably screen-positive prevalence and predictive value (PPV), MCADD prevalence and the proportion those diagnosed with MCADD in whom variants are classified as definite or uncertain clinical importance.

## METHODS

### Study population

Babies were screened at five to eight days of age^35^ in areas served by six newborn screening laboratories located in areas of high minority ethnic prevalence.^31^ Together they provide screening services to approximately 400,000 newborn infants each year, representing 60% of all births in England and Wales.^32^ These six laboratories use electrospray tandem mass spectrometry (MS/MS) to screen for phenylketonuria and have access to specialist clinical metabolic services. Standardized protocols were developed together with proformas for the prospective reporting of data. Screening for MCADD was not offered in other English laboratories during the pilot phase.

### Screening protocol

**Figure 1:**
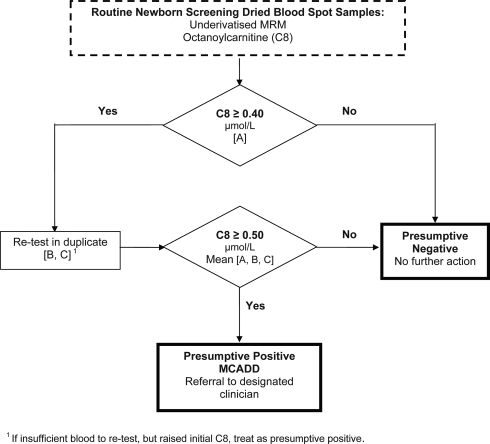
Screening protocol

Following midwife gained consent, a heel prick blood sample was collected onto Whatman or Schleicher and Schuell 903 (ID Biological Systems) filter paper cards as four dried blood spots (DBS). Laboratories analysed a single 3 mm punch without derivitization using MS/MS with multiple reaction mode (MRM) acquisitions for quantitation of octanoylcarnitine (C8), measured using an agreed single common screening protocol (Figure 1) with values ≥0.40 µmol/L repeated in duplicate using the same DBS sample. A presumptive positive screening result for MCADD was defined as an average triplicate C8 value ≥0.50 µmol/L. A full acylcarnitine profile was performed on presumptive positive samples.

### Diagnostic and clinical management protocols

All presumptive positive babies were referred for further diagnostic testing. Laboratory or clinical liaison staff contacted an assigned specialist paediatric metabolic team to arrange an emergency appointment for the family within 24 hours of the reported screening result. The family and their general practitioner (GP or family doctor) were contacted on the same day with the screening test result, information about MCADD, emergency contact numbers and the appointment details.

**Figure 2:**
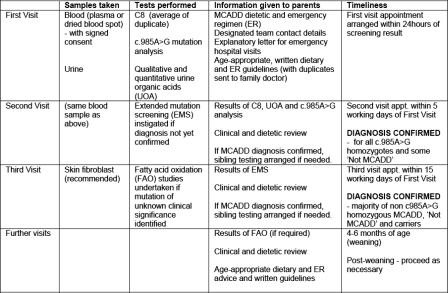
Diagnostic and clinical management schedule

Results of the full acylcarnitine profile from the screening sample were available at the first appointment. Clinicians followed an agreed diagnostic testing protocol and clinical management schedule as detailed in Figure 2. Initial diagnostic test results were available for parents within one week. Extended mutation screening (EMS) was arranged for all babies not homozygous for c.985A > G when associated with a persistent biochemical abnormality, defined as an average repeat C8 ≥ 0.50 µmol/L and/or abnormal qualitative urine organic acid (UOA) profile and/or quantitative urine organic acid (hexanoylglycine) ≥1.1 µmol/mmol creatinine.^36^ DNA was extracted from blood samples and PCR amplifications of all exons, including part of the flanking intron sequences of the human MCAD gene (*ACADM*) were carried out using intron-located primers under standard conditions. PCR fragments were sequenced on a 3100-Avant genetic analyzer using BigDye® Terminator v1.1 Cycle Sequencing kit (Applied Biosystems). We estimate that this strategy detects at least >95% of the causative mutations, since only two deletions have been described in the MCAD gene^37,38^ and several studies have demonstrated that gross genomic rearrangements in *ACADM* are very rare indeed.^3,39–43^

Fibroblast fatty acid oxidation (FAO) studies^44^ were recommended if mutations were identified of uncertain clinical significance.

Diagnostic and clinical management schedules for ‘at risk’ siblings included C8, qualitative UOA tests and genotyping once the proband diagnosis was confirmed.

### External Quality Assurance (EQA)

An EQA scheme was established, based at Birmingham Children's Hospital, providing a quality assessment of precision and accuracy for the analysis of C8 by MS/MS.^45^ Monthly specimens were circulated and results were analysed and reported back to all participating laboratories. Furthermore, a (quarterly) molecular EQA scheme was established for c.985A>G testing and monthly statistical summaries of population C8 values were produced to monitor between and within laboratory variation in population distributions.^46^

### Data notification and analysis

All presumptive positive screened babies were notified directly to the study co-ordinating centre by laboratory staff using an agreed standard proforma. Concurrently, paediatricians notified all newly diagnosed children with MCADD, including any diagnoses or deaths prior to screening, through the British Paediatric Surveillance Unit (BPSU).^47^ Notifications were followed up with questionnaires which were returned to the study co-ordinating centre. Data collected included initials, date of birth, partial postcode (for de-duplication), sex and ethnicity in addition to full screening and diagnostic test results and dates. Data were stored on a relational database (Microsoft Access 2003) and analysed using Stata IC version 10 (StataCorp, 2007).

Box 1 **MCADD of definite phenotype** (genotype of certain pathogenicity)

- persistent biochemical abnormality

 AND

- c.985A > G homozygous

 OR

- presence of two mutations, both either disease associated or predicting truncated protein

**MCADD of uncertain phenotype** (genotype of uncertain pathogenicity)

- persistent biochemical abnormality

 AND

- two mutations, at least one of whose pathogenicity unknown

 *NB. phenotype determined by the ‘milder’ mutation*

**Carrier**

- one mutation only (or with polymorphism)

 AND

- trace or no persistent biochemical abnormality

**Not MCADD**

- no mutations

 AND

- persistent biochemical abnormality but profile **not** indicative of MCADD

 OR

- no persistent biochemical abnormality

### Independent Diagnostic Review Panel (DRP)

An independent expert panel was convened to agree case definitions as a basis for assigning the outcome of screening and determining its validity. Four categories were agreed as shown in Box 1, with definite phenotype denoting clinically important diagnoses that have a strong probability of severe clinical presentation, and uncertain phenotype indicating genotypes not previously encountered amongst clinically presenting children or where a milder disease severity has been reported.

**Figure 3:**
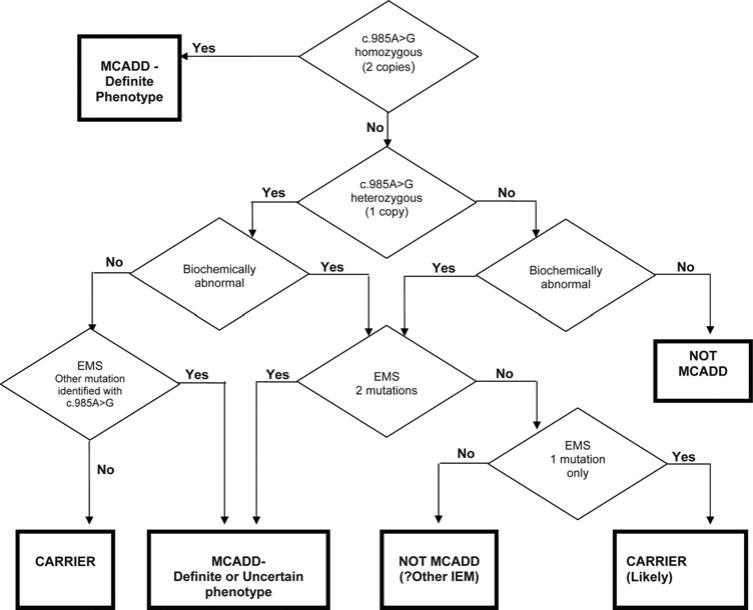
Diagnostic Review Panel (DRP) Decision Tree

The panel reviewed all screening and diagnostic results and relevant clinical information for babies with presumptive positive screening results, independently of actual clinical management, and assigned each to one of the four categories as outlined in Figure 3. The panel's final categorization was not relayed to the paediatrician.

### Ethics approval

The study was approved by the London Great Ormond Street Hospital for Children NHS Trust Multi-centre Research Ethics Committee (MREC) (Ref: 04/Q0508/2); and the Patient Information Advisory Group (subsequently the Ethics and Confidentiality Committee of the National Information Governance Board) (Ref: PIAG/BPSU-2-10(e)/2005).

## RESULTS

From March 2004 to February 2008, 1,568,445 babies (estimated 79% white, 10% Asian, 5% black^31^) were screened and 199 presumptive positive children referred; nine children were inappropriately referred (C8 value lower than 0.5 µmol/L). Of the remaining 190 babies, 90 were girls (48%, 1 missing), 157 (86%) were white, 16 (9%) were Asian, and 10 (5%) were from other (mixed/black/other) ethnic groups (7 missing), giving an overall screen positive prevalence of 1.2 per 10,000 (95% CI 1.1–1.4). The estimated screen positive prevalence amongst white and Asian babies was 1.3 (95% CI 1.1–1.5) and 1.0 (95% CI 0.6–1.7) per 10,000, respectively. All subsequent results relate to the 190 infants referred according to the agreed protocol.

In 169 babies (89%, 1 missing), the screening sample was taken between five to eight days with 13 (7%) taken before day five and seven (4%) after day eight. Mean age at sample analysis was 9.7 days (mode: 8 days; IQR: 8–11 days; 3 missing). Diagnostic blood samples were taken at a mean age of 12 days (mode: 11 days; IQR 10–13 days; 5 missing). The group assigned as ‘Not MCADD’ (*n* = 14) included five children with three other inborn errors of metabolism: multiple acyl-CoA dehydrogenase deficiency (MADD) (*n* = 3), pyruvate dehydrogenase (PDH) deficiency (*n* = 1) and carbamoyl phosphate synthetase (CPS) deficiency (*n* = 1), and nine children classified as false-positive including two premature infants (≤32 weeks gestation) both of whom died with cardiac and respiratory problems. Babies in this category were mostly identified by a full acylcarnitine scan (Table 1).

**Table 1:** Biochemical results by DRP category (*n* = 190)

		Screening C8 µmol/L	Diagnostic C8 µmol/L	Quantitative UOA (hexanoylglycine) µmol/mmol creatinine
	Number (%)	Median (Interdecile range)	Median (Interdecile range)	Median (Interdecile range)
MCADD – ALL	147 (77%)	1.63 (0.70–3.61)	1.36 (0.40–3.28)^5^	21.50 (5.40–37.00)^12^
MCADD – Definite phenotype	103 (54%)	1.88 (0.86–4.69)	1.72 (0.81–3.66)^4^	24.00 (13.40–40.00)^7^
c.985A > G homozygous	78	1.92 (0.86–4.69)	1.75 (0.99–3.75)^3^	24.10 (14.00–40.00)^6^
Other	25	1.81 (0.81–4.89)	1.40 (0.55–2.63)^1^	19.65 (7.50–40.00)^1^
MCADD – Uncertain phenotype	44 (23%)	0.87 (0.63–2.60)	0.86 (0.32–2.24)^1^	8.10 (1.50–26.90)^5^
Carrier	29 (15%)	0.62 (0.51–0.72)	0.23 (0.10–0.61)^1^	0.80 (0.40–2.00)^3^
Not MCADD	14 (7%)	0.72 (0.54–1.50)	0.25 (0.06–0.99)^3^	1.70 (0.40–48.40)^4^
Other conditions – MADD(3)				
PDH(1), CPS(1)	5	0.62 (0.57–1.71)	0.57 (0.50–1.15)^1^	16.75 (0.50–65.70)^1^
False-positive	9	0.73 (0.51–1.50)	0.13 (0.03–0.99)^2^	0.90 (0.40–3.00)^3^
All screen positive	190 (100%)	1.16 (0.59–3.18)	1.14 (0.15–3.09)^9^	16.50 (0.80–34.70)^19^

Superscript^N^ denotes number of missing observations

The prevalence of disease detected through screening was 0.94 per 10,000 babies screened (147/1,568,445, 95% CI: 0.79–1.10), giving a positive predictive value (PPV) of 77% overall (147/190, 95% CI: 71–83%), or 54% if only those with MCADD of a definite phenotype are included (103/190, 95% CI: 47–61%). Of those assigned as MCADD, 53% (78/147) were found to be homozygous for c.985A>G.

In babies of a definite clinical phenotype, 95% were white and 2% were Asian ([95/100; 95% CI 89–98; 3 missing], [2/100; 95% CI 0.1–7] respectively). For babies of uncertain phenotype, 67% were white and 28% were Asian ([29/43; 95% CI 52–80; 1 missing], [12/43, 95% CI 16–43] respectively). Seven of the 12 Asian babies were homozygous for c.946-6T>G (IVS10-6T>G) in the 3' splice site of exon 11 and one further Asian baby was heteroallelic for c.985A>G/c.946-6T>G. Amongst white and Asian infants, the overall PPV was 79% (124/157; 95% CI 72–85) and 88% (14/16; 95% CI 62–98) respectively. For MCADD of a definite phenotype alone, the PPV amongst white and Asian babies was 61% (95/157; 95% CI 53–68) and 13% (2/16; 2–38) respectively, whereas for MCADD of uncertain phenotype alone, the PPV was 18% (29/157; 95% CI 13–25) and 75% (12/16; 95% CI 48–93) for white and Asian babies respectively.

**Figure 4:**
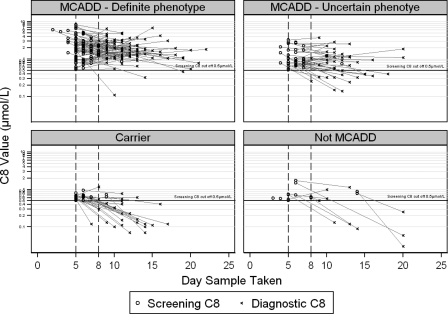
Screening and diagnostic C8 (µmol/L) by DRP category

Figure 4 shows screening and diagnostic C8 results for individual babies by day of sample taken and DRP category.

**Figure 5:**
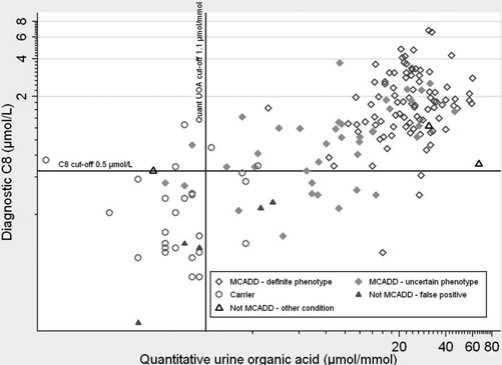
Diagnostic C8 (µmol/L) and quantitative UOA by DRP category

Figure 5 shows diagnostic C8 and quantitative urine organic acid results for individual babies by assigned DRP category.

The highest levels of C8 and quantitative UOA were observed amongst those assigned as MCADD of definite phenotype, particularly among those homozygous for c.985A > G.

**Figure 6:**
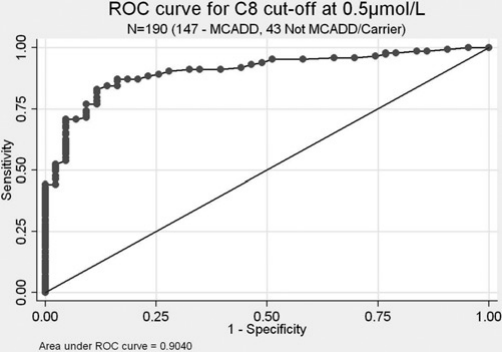
ROC curve for C8 cut off ≥0.5 µmol/L

Test accuracy was found to be high at C8 ≥ 0.5 µmol/L with an area under ROC curve (Figure 6) of 0.904 compared with 0.762 at C8 ≥ 0.8 µmol/L (not shown). This reflected the wide distribution of C8 screening results in babies confirmed as carriers and not MCADD.

Four babies were diagnosed before screening, of whom one died at three days of age and three became clinically unwell between days two and four. Furthermore, 12 babies with a previously diagnosed older sibling were tested and diagnosed early. With the addition of these 16 diagnoses, the prevalence of MCADD is 1.0 per 10,000 (163/1,568,445). Five additional MCADD diagnoses were made amongst older siblings in four families as a consequence of a newly diagnosed screened infant, three of definite and two of uncertain phenotype. No reports of clinically presenting (false-negative) MCADD cases were ascertained within the first two years of life through concurrent surveillance in this screened cohort.

## DISCUSSION

We report findings from a large-scale prospective study of newborn screening for MCADD conducted within a multi-ethnic population where over 20% of the population is non-white, principally Asian or black. This multicentre collaboration across six centres used common protocols for screening and diagnosis and developed a process by which screen positive cases were independently reviewed to assign MCADD diagnoses and infer their underlying clinical severity.

Approximately 1 in 10,000 screened babies were diagnosed with MCADD, approximately 60 per year in England. In the majority, the biochemical and genetic features were consistent with a high risk of clinical severity. The numbers of children with false-positive or carrier diagnoses were minimal reflecting prospective design features, namely thresholds selected for referral as well as the decision to restrict genetic testing to those referred. High specificity and overall PPV were achieved – a key requirement of screening programmes, demonstrating that the majority of babies who were detected were likely to benefit from screening.

Babies who were assigned with a phenotype of definite clinical importance were predominantly of white ethnic origin, whereas nearly a third of those of uncertain phenotype were Asian. Whilst recognizing that the number of Asian babies was small, these marked differences were reflected in the estimated PPV for phenotypes of definite and uncertain clinical importance amongst those of white and Asian ethnicity. Of particular note were seven babies homozygous for c.946-6T>G (IVS10-6T>G). In our study, this mutation was only found amongst Asian babies and has not previously been reported.

Particular strengths of this study include its large scale, adherence to common protocols and high completeness of data. In addition, we developed a novel classification system allowing clinical severity to be inferred, and appropriate for use within a screening programme in which affected children are, in most cases, asymptomatic and/or without family history. By categorizing babies this way, a marker for measuring clinical validity of the screening programme was established.

A limitation of our study is that clinical significance, outcome and benefit for babies assigned as MCADD of uncertain phenotype is not known, since almost all babies identified through newborn screening are currently treated, irrespective of the likely severity of their disease. Thus, the natural history of novel mutations detected only through screening remains difficult to assess. No false-negative diagnoses were reported from this cohort over a two-year follow-up, but longer-term follow-up is required.

We compare our data, as the largest such study published worldwide, with recent, international studies which have reported on the incidence, rate of MCADD diagnoses and number of c.985A>G homozygotes detected through newborn screening, summarized in Table 2, updating recently published reports.^2,12,48^

**Table 2:** Estimated incidence of MCADD through newborn screening

Area	No. Screened	No. MCADD	Incidence (per 100,000)	Rate	No. c.985A > G HMZ (% of all MCADD)
England (pilot screening study)	1,568,445	147	9.4	1/10,700	78 (53%)
Canada (Ontario)^48^	439,000	31	7.1	1/14,000	15 (48%)
Australia (NSW)^16^	461,500	24	5.2	1/19,200	12 (50%)
USA (NY State)^26^	385,893	20	5.2	1/19,300	6 (30%)
Germany (Bavaria)^25^	470,247	58	12.3	1/8,100	24 (41%)
Netherlands^24^	66,216	14	21.1	1/6,600	10 (71%)

Early neonatal presentation and death due to MCADD, typically occurring around two to three days of age, has been reported, but is uncommon and unlikely to be prevented by newborn screening, given the timing of bloodspot sample, analysis and reporting.^7,9,18,49^ In the UK, bloodspot samples for newborn screening are taken between five to eight days of age, later than in many countries.^16,24,25,41^ However, even within screening progammes which sample blood earlier, typically between 48–72 hours, preventative action would be unlikely as the screening result is reported later. We therefore found no evidence in our study to suggest that a change in UK screening policy to earlier bloodspot sampling would prevent decompensation or death in this early neonatal period.

The C8 cut-off set within England is lower than in many countries which screen at an earlier age and we acknowledge that the chosen cut-off must be appropriate for the population in question and the age at which screening takes place. We have recently examined C8 concentrations in the first two weeks of life^46^ in an analysis of newborn screening data for 227,098 infants from England and New South Wales, Australia (average postnatal age at testing of 5 and 3 days respectively) in which we demonstrated that median C8 concentrations do not vary significantly by age of sampling in unaffected babies between day three and day 14. Thus the cut-off in our study would be relevant to samples collected over this age range.

MCADD dietary guidelines and other information for parents and professionals, developed specifically through this study, have worked well and are freely available for parents and health-care professionals, along with current UK Standards and Guidelines for Newborn Bloodspot Screening from the UK Newborn Screening Programme Centre, at www.newbornbloodspot.screening.nhs.uk.

For most babies, assignment of the diagnostic category through the independent review process was straightforward. However, in a small number this proved difficult. In two babies, both ultimately assigned as carriers, a second mutation (in conjunction with c.985A>G) of unknown clinical significance (c.387 + 40G>A [or IVS5 + 40G>A] and c.388-9C>T [or IVS5-9C>T]) was identified but no biochemical abnormality was detected at diagnosis, including one with normal FAO studies. Furthermore, five babies, also assigned as carriers (simple c.985A > G heterozygotes), showed elevated diagnostic C8 (range 0.54–1.17 µmol/L) despite having no second mutation identified at EMS and no other biochemical abnormality detected including one with normal FAO studies. These issues illustrate the difficulties faced by paediatricians when deciding whether to treat a child with borderline results or only some of the biomarkers associated with MCADD. Whilst completely independent, we are not aware of any discrepancies between assigned classification and the final clinical management of the child.

Nine babies were inappropriately referred with an average C8 result between 0.4 and 0.5 µmol/L. Diagnostic testing revealed three carriers, four ‘Not MCADD’ and two assigned as MCADD of uncertain phenotype. Of the latter two, one had no biochemical abnormalities detected at diagnostic testing and was found to be heteroallelic for c.985A>G and c.127G>A. Previous studies note that this genotype does not express the biomarkers normally associated with MCADD.^5,40,41,43^ The second case was found to be heteroallelic for c.985A>G and c.199T>C, and, although both C8 results fell below the 0.5 µmol/L cut-off, some organic acid abnormalities were detected. We acknowledge that by strict adherence to an agreed C8 cut off, there may occasionally be cases of (mild) MCADD which are not detected through screening. However, screening is not diagnosis and, by reviewing and reporting these findings, we are able to conclude that at least seven of these nine referrals were made unnecessarily.

Very few carriers were detected in relation to expected total carrier pool. Carrier frequency for c.985A>G in the white UK population has been estimated at 1/65 (95% CI 1/71–1/61)^31^ assuming Hardy Weinberg equilibrium. Within our screened study population (*n* = 1,568,445), an estimated 79.5% or 1,246,914 were white, of which 19,183 (or 1.5%) would be anticipated c.985A>G carriers.^31^ In total, 23 white, c.985A>G heterozygote carriers were detected over the four-year study, 0.1% of the expected frequency. Moreover, we expect that the number of carriers identified through newborn screening for MCADD to be further reduced, as of April 2010, by the introduction within the screening protocol of an additional C8/C10 ratio measurement with a referral cut off ≥1.0. Use of the C8:C10 ratio has been shown to discriminate between MCADD phenotypes well.^41,50–52^ Screen positive MCADD referrals in England will now only be made if C8 ≥ 0.5 µmol/L and C8/C10 ≥ 1.0. We have estimated through a specific research audit within this study (results not presented here), that the detection of carriers and those assigned as ‘Not MCADD’ will be reduced by over 90%, minimizing unnecessary referral and anxiety for families and increasing the predicted PPV at screening for all MCADD phenotypes to over 95%.

Our findings demonstrate that prospectively defined, quality assured screening and diagnostic protocols allow identification of children with clinically important disease whilst minimizing the harms of screening related to the detection of children with biochemical or genetic variations of uncertain prognostic significance.

In 2006, interim data from this study were reviewed by the UK National Screening Committee and, as a result, MCADD screening was successfully implemented across England by April 2009.

## CONCLUSION

Newborn screening identifies MCADD in 1/10,000 babies born in England. In the majority, the features indicate a phenotype of definite clinical importance, suggesting that the validity of newborn screening is high. Ethnic variations in genotype and phenotype were noted. Diagnostic protocols need to reflect the ethnic diversity of the contemporary English population. This prospective collaborative study supplied evidence and informed the policies for a high-quality and rapidly implemented screening programme, and further enhances the information available for evaluating newborn screening for rare disorders in multi-ethnic populations.

**Abbreviations:** MCADD – Medium chain acyl-CoA dehydrogenase deficiency; DRP – Diagnostic Review Panel; EMS – Extended Mutation Screening; FAO – Fatty acid oxidation; ER – Emergency regimen; DBS – Dried blood spot; UOA – Urine organic acid; C8 – Octanoylcarnitine; PPV – Positive predictive value; EQA – External quality assurance; MS/MS – Tandem mass spectrometry
